# Screening for Autism Spectrum Disorders with the Brief Infant-Toddler Social and Emotional Assessment

**DOI:** 10.1371/journal.pone.0097630

**Published:** 2014-05-22

**Authors:** Ingrid Kruizinga, Janne C. Visser, Tamara van Batenburg-Eddes, Alice S. Carter, Wilma Jansen, Hein Raat

**Affiliations:** 1 Department of Public Health, Erasmus University Medical Center, Rotterdam, the Netherlands; 2 Karakter University Center Nijmegen, Nijmegen, the Netherlands; 3 Department of Psychology and Education, VU University, Amsterdam, the Netherlands; 4 Department of Psychology, University of Massachusetts Boston, Boston, Massachusetts, United States of America; 5 Department of Youth Policy, Rotterdam Municipal Health Service (GGD Rotterdam-Rijnmond), Rotterdam, the Netherlands; UNC Chapel Hill, United States of America

## Abstract

**Objective:**

Using parent-completed questionnaires in (preventive) child health care can facilitate the early detection of psychosocial problems and psychopathology, including autism spectrum disorders (ASD). A promising questionnaire for this purpose is the Brief Infant-Toddler Social and Emotional Assessment (BITSEA). The screening accuracy with regard to ASD of the BITSEA Problem and Competence scales and a newly calculated Autism score were evaluated.

**Method:**

Data, that was collected between April 2010 and April 2011, from a community sample of 2-year-olds (N = 3127), was combined with a sample of preschool children diagnosed with ASD (N = 159). For the total population and for subgroups by child's gender, area under the Receiver Operating Characteristic (ROC) curve was examined, and across a range of BITSEA Problem, Competence and Autism scores, sensitivity, specificity, positive and negative likelihood ratio's, diagnostic odds ratio and Youden's index were reported.

**Results:**

The area under the ROC curve (95% confidence interval, [95%CI]) of the Problem scale was 0.90(0.87–0.92), of the Competence scale 0.93(0.91–0.95), and of the Autism score 0.95(0.93–0.97). For the total population, the screening accuracy of the Autism score was significantly better, compared to the Problem scale. The screening accuracy of the Competence scale was significantly better for girls (AUC = 0.97; 95%CI = 0.95–0.98) than for boys (AUC = 0.91; 95%CI = 0.88–0.94).

**Conclusion:**

The results indicate that the BITSEA scales and newly calculated Autism score have good discriminative power to differentiate children with and without ASD. Therefore, the BITSEA may be helpful in the early detection of ASD, which could have beneficial effects on the child's development.

## Introduction

Preventive child health care offers a systematic opportunity for the early detection of psychosocial problems and psychopathology, such as autism spectrum disorders (ASD), among toddlers. In the Netherlands, preventive child health care for children of ages 0–4 years is delivered through community well-child clinics that provide routine developmental assessment and vaccinations (i.e. well-child visits) and that are free of charge [1].

ASD represents a set of neurodevelopmental disorders that are characterized by impairments in the domains of reciprocal social interactions and communication and by restrictive, stereotyped patterns of behavior [2]. In the current Diagnostic and Statistical Manual of Mental disorders, 5^th^ edition, ASD's are part of the pervasive developmental disorders and classified into three main categories, namely: autistic disorder, Asperger's disorder and pervasive developmental disorder-not otherwise specified [2]. Studies report ASD prevalence rates of about 1.0% [3], [4]. Abnormal functioning that is indicative of ASD starts before 3 years of age [2]. On average, the first symptoms to arouse parental concerns about children eventually diagnosed with ASD occur before the second birthday. However, the average age of ASD diagnosis is approximately three years of age and often occurs later [5]. These findings suggest that it should be possible to detect and diagnose ASD earlier. Early detection of ASD is important because early access to interventions may improve children's outcomes, [6], [7] and diagnosis may enhance parent's understanding and coping with the impairments of their child [8].

One approach for facilitating early identification of ASD is the population-based screening of children as part of well-child visits using parent-completed questionnaires [9], [10] Several instruments are developed for the early detection of ASD, of which the use of the Checklist for Autism in Toddlers (CHAT) [11] and the Modified Checklist for Autism in Toddlers (M-CHAT) [12] is advocated by autism support organizations [13]. However, early detection instruments that are used in a preventive health care setting should cover a broad range of psychosocial problems, since limited time and capacity in the preventive child health care make it undesirable to screen for each psychosocial problem separately. Also, it has been shown that psychosocial problems tend to co-occur, [14], [15] and that individual problems may apply to more than one disorder [16]. In addition to measuring problem domains, it is crucial to also measure competence domains. Delays in the acquisition of competencies are strongly related to a wide range of psychosocial problems later in life [17] and are often the prodromal signs of developmental disorders, such as ASD [18].

The Brief Infant-Toddler Social and Emotional Assessment (BITSEA) [19] is a promising and short (42 items) questionnaire, that measures both problems (Problem scale) and delays in the acquisition of competencies (Competence scale) in 1–3 year olds, and also consists of items designed to measure ASD symptoms. The BITSEA is not designed to diagnose ASD, but it may be useful as a screener for identifying children with this disorder [20]. Previous studies have shown that the BITSEA Problem and Competence scale has adequate reliability for the Problem scale and validity for the Problem and Competence scale [19], [21]–[23]. The study performed in the Netherlands [23] evaluated among others the internal consistency, test-retest reliability, concurrent validity and discriminant validity. An adequate Cronbach's alpha (i.e. >0.70[24]) was found for the Problem scale (0.76) and marginal for the Competence scale (0.63). Test-retest reliability was adequate (>0.70 [25]) for the Problem scale (0.75) and marginal for the Competence scale (0.61). The BITSEA Problem scale was positively correlated with the CBCL, Pearson coefficients of 0.66 (Internalizing), 0.65 (Externalizing) and 0.75 (Total Problem). The BITSEA Competence score was negatively correlated with the CBCL, Pearson coefficients of −0.26 (Internalizing), −0.23 (Externalizing) and −0.26 (Total Problem). All correlations were significant (p<0.01). The mean BITSEA score was compared between a group of parents that worried about the development of their child and a group that did not worry. The Problem and Competence score were significantly less favourable in the group of parents that worried, compared to the group of parents that did not worry (effect sizes were respectively 0.93 and 0.52).”

Also the sensitivity and specificity of the BITSEA has been evaluated in several studies [19], [26], [27] One study, conducted in the United States [19], examined its sensitivity and specificity in a community sample of 1280 children. In this study, children with scores in the clinical range on the Child Behavioral Checklist (CBCL1.5-5) [28] and Infant-Toddler Social and Emotional Assessment (ITSEA) [29], [30] were used as reference groups for the evaluation of the Problem scale. A sensitivity of respectively 93.2% and 78.1% and a specificity of respectively 78.0% and 88.8% were found. The Competence scale was evaluated against a group of children with a score in the clinical range on the ITSEA and had a sensitivity of 68.9% and a specificity of 95.1%. Problem scale cutpoints were chosen at scores of ≥75^th^ percentile and Competence scale cutpoints were chosen at scores of <15^th^ percentile [31]. In a Turkish study [26], in a community sample of 462 children, sensitivity and specificity of only the Competence scale was examined relative to children treated in a child psychiatry outpatient clinic with an autism diagnosis (n = 35). In this study, the sensitivity was 72%–93% and specificity was 76%–85%, depending on the cutpoint chosen. A Dutch study [27] evaluated the screening accuracy of the BITSEA Problem scale more extensively than prior studies. The screening accuracy was evaluated with multiple indices (i.e. area under the curve, sensitivity, specificity, likelihood ratio's, diagnostic odds ratios and Youden's index) by calculating receiver operating characteristic (ROC) curves of the BITSEA Problem scale relative to the CBCL Total Problem scale. Indices of screening accuracy for a range of BITSEA Problem scores were presented, because different cutpoints might be chosen in different settings (e.g. clinical application versus epidemiological research). In that study, the screening accuracy of the BITSEA Competence scale was not evaluated with a reference group of children with a CBCL Total Problem score in the clinical range, since the CBCL Total Problem score does not measure competencies.

In the present study we aim to evaluate the screening accuracy of both the BITSEA Problem and Competence scales with regard to an ASD diagnosis. Additionally, we will evaluate the screening accuracy of the BITSEA items that are specifically intended to signal ASD, since little is known about the performance of these items in the detection of ASD. Previous studies showed differences in mean BITSEA scores between boys and girls (with boys scoring less favourably) [19], [22], [23], therefore the screening accuracy is also evaluated in subgroups by child gender.

## Method

### Ethics Statement

Regarding the data collection of the community sample; only anonymous data were used and the questionnaires were completed on a voluntary basis by the parents. Parents received written information on these questionnaires and were free to refuse to participation. Observational research with data does not fall within the ambit of the Dutch Act on research involving human subjects [32] and does not require the approval of an ethics review board. The Medical Ethics Committee of the Erasmus Medical Centre Rotterdam declared to have no objection (‘formal waiver’) regarding the study protocol and consent procedures. The Medical Ethical Committee of the University Medical Centre St. Radboud Nijmegen approved the study protocol regarding the ASD-study. We are prepared to make the data available upon request.

### Design and participants

For the present study, data from two separate samples were combined. First, data from a community sample of 2-year old children was used. These data were gathered between April 2010 and April 2011 by child health care organizations in the context of routine health examinations in the Rotterdam area, the Netherlands. Parents of 3170 children that attended the well-child visit handed in the questionnaire (95.5% of all parents that attended the well-child visit). Children were excluded from the analyses if there were too many missing items on both BITSEA scales [20] (n = 43), leaving a study population of 3127 (94.2%) children. No children in the community sample were under treatment of a mental health professional at the time of inclusion. Details on the design and participants of the community sample are described elsewhere [23].

Second, data from a sample of children diagnosed with ASD were used (i.e. ASD-sample). Children between the ages of 12–40 months were recruited in the DIANE-study (Diagnosis and Intervention of Autism in the Netherlands) [33] at Karakter Child and Adolescent Psychiatry University Center Nijmegen, the Netherlands. Children with a positive score on the Early Screening of Autistic Traits Questionnaire [34] and/or for whom there were major concerns regarding social and communicative development entered the study between spring 2004 and spring 2007. Parents of the ASD-sample completed the ITSEA (i.e. a more comprehensive measure that includes the BITSEA items) at home before their first visit for diagnostic assessments and all children underwent an extensive psychiatric assessment (i.e. administration of the Autism Diagnostic Observation Schedule and Autism Diagnostic Interview-Revised) observations of standardised parent-child play and standardised assessment of cognitive and language skills). Details on the design and participants of the ASD-sample are described elsewhere [35]. For the purpose of this study, answers on BITSEA items were extracted from the larger pool of ITSEA items. Children were excluded from the analyses if they did not receive a diagnosis (n = 29), if they received a diagnosis other than ASD (n = 69) (i.e. false positives), if there were too many missing items on the BITSEA scales [20] (n = 19), or if they were younger than 12 months (n = 2) leaving a study population of 159 (57%) children.

### Measures

The BITSEA, designed for 1-to-3-year old children, consists of 42 items with three response options (‘not true/rarely’(0), ‘somewhat true/sometimes’(1), ‘very true/often’(2)) and comprises two multi-item scales; a Problem scale (31 items) and a Competence scale (11 items). The Problem scale assesses social-emotional/behavioral problems such as aggression, defiance, overactivity, negative emotionality, anxiety, and withdrawal. The Competence scale assesses social-emotional abilities such as empathy, prosocial behaviors, and compliance [31]. Responses can be summed for each scale: a high score on the Problem scale and/or a low score on the Competence scale is less favourable [20]. The BITSEA also consists of 17 items that are specifically included for the early detection of ASD belonging to either the Problem scale (9 items) or the Competence scale (8 items). The autism items reflect problems behaviors that are typical of children with ASD (e.g. *put things in a special order over and over*) and competencies in which deficits are often present in children with ASD (e.g. *points to show you something far away*) [20]. Although these items formally do not represent a separate scale, we calculated the Autism score analogous to the Problem scale score, yielding a good internal consistency (Cronbach's alpha = 0.77). Answers on the autism items belonging to the Competence scale were first reversed before all autism items were summed, so a higher Autism score would represent more problems and fewer competencies. Children with more than 3 missing items were excluded for analyses (n = 48). Excluded children were all part of the community sample.

Items on standard socio-demographic variables were included: child age and gender.

### Analyses

#### Demographic characteristics and mean BITSEA scores

Differences in mean BITSEA scores and child age between the community sample and the ASD-sample were tested with independent sample t-tests. Differences in gender composition of the community sample and ASD-sample were tested with Chi-square tests.

#### Screening accuracy

Screening accuracy was evaluated by calculating receiver operating characteristic (ROC) curves, with a reference group that consists of children with a diagnosis of ASD. The area under the ROC curve was examined, along with, for a range of Problem and Competence scale scores and the Autism score; sensitivity, specificity, positive test likelihood ratio (LHR^+^) and negative test likelihood ratio (LHR*^−^*), diagnostic odds ratio (OR) and Youden's index. All indices for screening accuracy were evaluated for the total sample as well as for boys and girls separately.

The ROC curve is a plot of sensitivity as a function of 1-specificity for all possible cutpoints of the BITSEA. The greater the area under the curve (AUC), the more discriminative power the BITSEA has in differentiating children with and without ASD. An AUC>0.90 indicates high accuracy; 0.70≤AUC<0.90 indicates moderate accuracy; 0.50≤AUC<0.70 indicates low accuracy; and AUC = 0.50 is chance level accuracy [36]. We examined the 95% confidence intervals of the AUCs to evaluate whether the screening accuracy differed significantly between subgroups.

To determine the optimal cutpoint, the Youden index was used, which is defined as the maximum vertical distance between the ROC curve and the diagonal or chance line and is calculated as *Youden's index = sensitivity+specificity-1* [37].

Sensitivity is the proportion of true positives that are correctly identified by the test; specificity is the proportion of true negatives that are correctly identified by the test. To further investigate the correctness of classification, likelihood ratios were calculated. *LHR^+^ = sensitvitiy/(1-specificity)* is the ratio of the probability of a positive test result if the outcome is positive (true positive) to the probability of a positive test result if the outcome is negative (false positive); *LHR^−^  = (1-sensitivity)/specificity* is the ratio of the probability of a negative test result if the outcome is positive (false negative) to the probability of a negative test result if the outcome is negative (true negative). LHR^+^>7.00 and LHR*^−^*<0.30 indicate high screening accuracy [38].

The OR = sensitivity*specificity/((1-sensitivity)*(1-specificity)) = LHR^+^/LHR^−^ of a test is the ratio of the odds of a positive test result when having the ‘disorder’ relative to the odds of a positive test result when not having the ‘disorder’. The values of OR ranges from zero to infinity, with higher values indicating better discriminatory test performance. OR>20.00 indicate high screening accuracy [38].

The AUC, Youden's index, sensitivity, specificity, LHR^+^, LHR*^−^* and OR are independent of prevalence of the ‘disorder’, as opposed to the positive predictive value and negative predictive value, therefore the latter were not evaluated in this study. [38].

All analyses were performed in SPSS 20.0 (SPSS Inc. 2011).

## Results

The demographic characteristics of the multiethnic community sample and ASD-sample are presented in Table 1. In comparison to the community sample, the ASD-sample consisted of older children (*t* = 58.3, *p*<0.001) and more boys (*Χ^2^* = 50.2, *p*<0.001).

**Table 1:** Child characteristics of the autism spectrum disorder (ASD) sample and community sample.

	ASD-sample N = 159	Community sample N = 3127
	**Percentage (N)**	**Percentage (N)**
*Gender^a^* *		
boys	79.2 (126)	50.0 (1564)
girls	20.8 (33)	49.1 (1535)
	**Mean (SD)**	**Mean (SD)**
Age (months)*	31.8 (6.4)	23.7 (0.7)
BITSEA Problem scale score*	20.5 (8.7)	7.8 (5.3)
BITSEA Competence scale score*	10.0 (4.0)	17.5 (3.0)
BITSEA Autism score*	14.6 (5.2)	4.1 (3.3)

a. Percentages do not sum to 100% due to missing values.

* Significant differences in composition between ASD-sample and community sample with regard to gender, and age and mean Problem scale score, Competence scale score, and Autism score, *p*<0.001.

### Mean BITSEA scores

The mean Problem and Competence scale scores and the Autism score are presented in Table 1. In comparison to children in the community sample, children in the ASD-sample scored less favourably on the Problem scale (*t* = 28.1, *p*<0.001), the Competence scale (*t* = 29.9, *p*<0.001) and Autism score (*t* = 37.3, *p*<0.001).

### Screening accuracy

**Figure 1:**
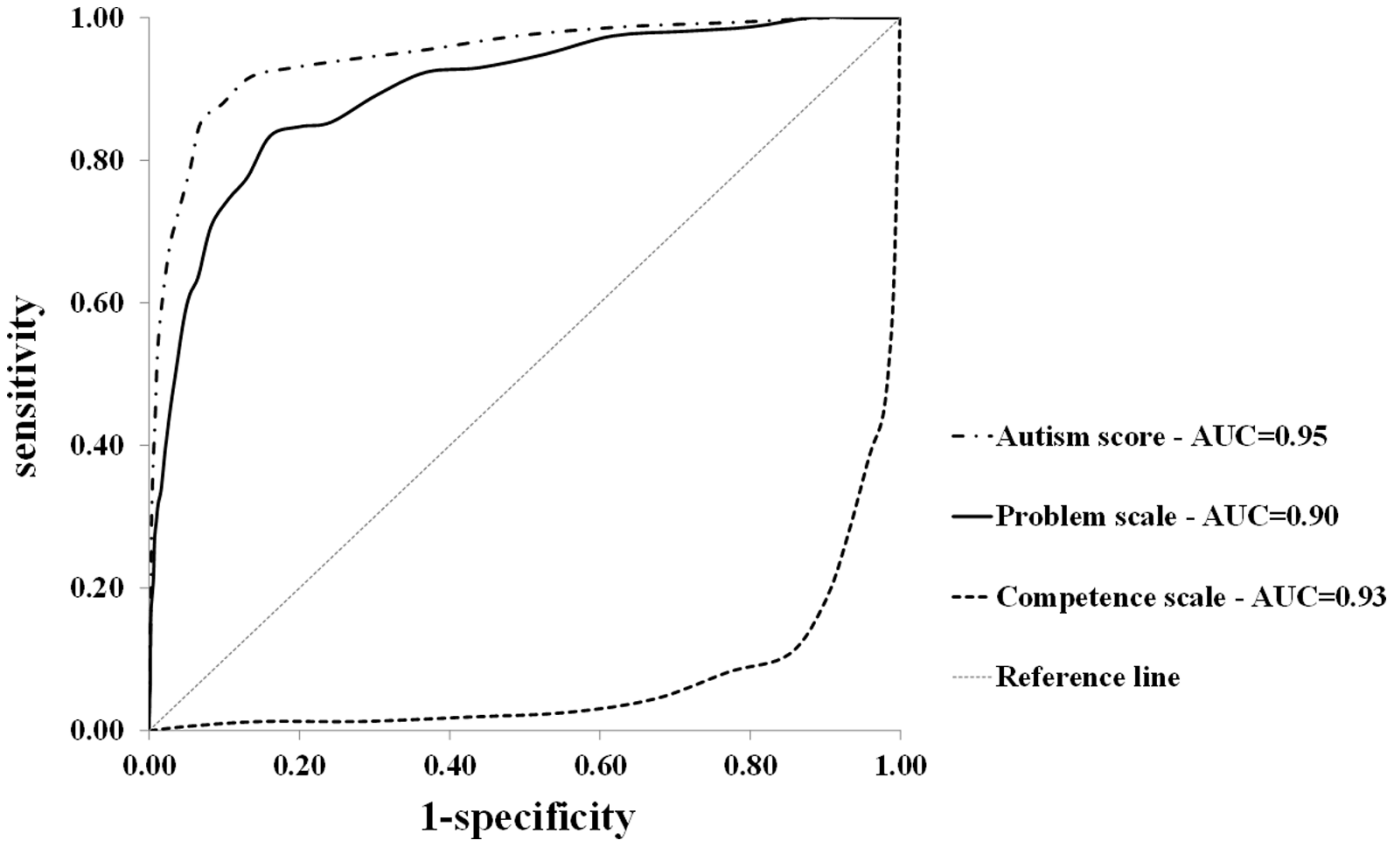
ROC curves and AUC of the BITSEA Problem and Competence scale and BITSEA Autism score relative to a sample of children with a diagnosis of autism spectrum disorder.

ROC curves of the Problem and Competence scale scores and Autism score are presented in Figure 1. In Table 2, the AUC and sensitivity, specificity, LHR^+^, LHR*^−^*, OR and Youden's index are presented for a range of BITSEA scale, for the total population and for subgroups by child gender.

**Table 2:** The screening accuracy of the BITSEA scales with regard to autism spectrum disorders: Area Under the Curve and sensitivity, specificity, liklihood ratio's, diagnostic odd ratio and Youden's index for a range of Problem and Competence scores, for the total sample and for subgroups by gender.

Scale	BITSEA Problem							BITSEA Competence						
Total	AUC = 0.90 (95% CI = 0.87–0.92)							AUC = 0.93 (95% CI = 0.91–0.95)						
N = 3286	score	sens	spec	LHR^+^	LHR*^−^*	OR	J	score	sens	spec	LHR^+^	LHR*^−^*	OR	J
	9	0.92	0.63	2.51	0.12	20.73	0.56	11	0.98	0.56	2.20	0.04	52.97	0.53
	10	0.89	0.70	2.94	0.16	18.92	0.59	12	0.96	0.61	2.48	0.07	37.00	0.57
	11	0.85	0.76	3.53	0.19	18.24	0.61	13	0.93	0.72	3.34	0.10	35.01	0.65
	12	0.85	0.80	4.22	0.19	22.05	0.65	14	0.90	0.82	4.91	0.12	40.41	0.72
	**13**	**0.83**	**0.84**	**5.18**	**0.20**	**26.22**	**0.67**	**15**	**0.85**	**0.89**	**7.92**	**0.17**	**47.95**	**0.74**
	14	0.78	0.87	5.92	0.26	23.06	0.65	16	0.77	0.92	9.38	0.25	37.71	0.69
	15	0.75	0.89	7.08	0.28	24.85	0.64	17	0.67	0.96	15.19	0.34	44.37	0.63
	16	0.71	0.92	8.60	0.32	26.93	0.62	18	0.56	0.97	21.95	0.46	48.15	0.53
	17	0.64	0.93	9.75	0.39	25.10	0.57	19	0.43	0.98	22.48	0.58	38.49	0.41
**Boys**	AUC = 0.88 (95% CI = 0.85–0.91)							AUC = 0.91 (95% CI = 0.88–0.94)*						
**N = 1690**	9	0.92	0.60	2.31	0.13	17.48	0.52	11	0.97	0.53	2.08	0.05	42.04	0.51
	10	0.88	0.67	2.64	0.18	14.80	0.55	12	0.95	0.60	2.34	0.09	27.19	0.54
	11	0.85	0.73	3.19	0.21	15.50	0.58	13	0.92	0.71	3.12	0.12	26.66	0.62
	12	0.84	0.78	3.88	0.20	19.17	0.62	14	0.88	0.82	4.84	0.14	33.88	0.70
	**13**	**0.83**	**0.82**	**4.61**	**0.20**	**22.63**	**0.65**	**15**	**0.82**	**0.88**	**6.92**	**0.20**	**34.70**	**0.71**
	14	0.77	0.85	5.18	0.27	19.18	0.62	16	0.73	0.90	7.65	0.30	25.51	0.63
	15	0.74	0.88	6.02	0.30	20.17	0.62	17	0.62	0.94	11.23	0.40	28.23	0.57
	16	0.70	0.90	7.28	0.33	21.81	0.60	18	0.50	0.97	15.66	0.52	30.15	0.47
	17	0.63	0.92	8.06	0.40	19.92	0.55	19	0.37	0.98	15.54	0.65	24.09	0.35
**Girls**	AUC = 0.93 (95% CI = 0.89–0.97)							AUC = 0.97 (95% CI = 0.95–0.98) *						
**N = 1568**	9	0.94	0.66	2.79	0.10	28.74	0.60	11	0.98	0.66	2.85	0.03	91.70	0.64
	10	0.94	0.73	3.46	0.09	39.10	0.66	12	0.97	0.69	3.11	0.04	72.11	0.66
	11	0.87	0.79	4.05	0.16	24.65	0.66	13	0.95	0.78	4.32	0.07	62.63	0.73
	12	0.87	0.82	4.79	0.16	30.36	0.69	14	0.92	0.81	4.90	0.10	49.22	0.73
	**13**	**0.84**	**0.86**	**6.04**	**0.19**	**32.24**	**0.70**	**15**	**0.88**	**0.94**	**14.14**	**0.12**	**113.81**	**0.82**
	14	0.81	0.89	7.22	0.22	33.11	0.69	16	0.82	0.97	26.15	0.19	138.50	0.79
	15	0.77	0.91	8.94	0.25	36.14	0.69	17	0.73	1.00	x	0.27	x	0.73
	16	0.74	0.93	10.89	0.28	39.33	0.67	18	0.62	1.00	x	0.38	x	0.62
	17	0.68	0.95	12.97	0.34	38.09	0.63	19	0.49	1.00	x	0.51	x	0.49

* The Competence scale AUCs differ significantly between boys and girls (i.e. the 95% confidence intervals do not overlap)

Note: AUC = area under the curve; 95%CI = 95% confidence interval; sens = sensitivity; spec = specificity; LHR^+^ = likelihood

ratio positive test; LHR*^−^* = likelihood ratio negative test; OR = diagnostic odds ratio; J = Youden's index.

All AUC's were significant (p<0.001). Scores with the highest unrounded Youden's index are indicated in bold.

The AUC's (95% confidence interval [CI]) of the Problem scale was 0.90(0.87–0.92), and of the Competence scale 0.93(0.91–0.95). The screening accuracy of the Problem scale was equal for girls (AUC = 0.93; 95%CI = 0.89–0.97) and boys (AUC = 0.88; 95%CI = 0.85–0.91). The screening accuracy of the Competence scale was better for girls (AUC = 0.97; 95%CI = 0.95–0.98) than for boys (AUC = 0.91; 95%CI = 0.88–0.94). The Youden index indicated the same optimal cutpoint for the total population and for boys and girls for the Problem scale (score 13) and for the Competence scale (score 15).

In Table 3 AUCs and sensitivity, specificity, LHR^+^, LHR*^−^*, OR and Youden's index are presented for a range of Autism scores for the total population and for subgroups by child gender. The AUC was 0.95(0.93–0.97) and the screening accuracy was equal for girls (AUC = 0.97; 95%CI = 0.95–0.99) and boys (AUC = 0.93; 95%CI = 0.91–0.96). The Youden index indicated different optimal cutpoint for the total population (score 10) and for boys (score 9) and girls (score 8).

**Table 3:** The screening accuracy of the BITSEA Autism score: Area Under the Curve and sensitivity, specificity, liklihood ratio's, diagnostic odds ratio and Youden's index for a range of Autism scores, for the total sample and for subgroups by gender.

	BITSEA Autism score						
Total	AUC = 0.95 (95% CI = 0.93–0.97)						
**N = 3236**	**score**	**sens**	**spec**	**LHR^+^**	**LHR** *^−^*	**OR**	**J**
	6	0.94	0.72	3.43	0.08	43.33	0.67
	7	0.93	0.81	4.86	0.09	56.11	0.74
	8	0.92	0.86	6.77	0.10	70.71	0.78
	9	0.88	0.90	9.05	0.13	67.53	0.78
	**10**	**0.85**	**0.93**	**12.40**	**0.16**	**78.79**	**0.78**
	11	0.79	0.95	14.39	0.22	64.70	0.73
	12	0.72	0.96	19.44	0.29	66.80	0.68
	13	0.68	0.97	25.35	0.33	75.96	0.65
	14	0.59	0.98	37.59	0.42	89.38	0.57
**Boys**	AUC = 0.93 (95% CI = 0.91–0.96)						
**N = 1671**	5	0.94	0.59	2.29	0.09	24.30	0.53
	6	0.94	0.70	3.08	0.09	33.74	0.63
	7	0.92	0.78	4.10	0.10	40.05	0.70
	8	0.90	0.84	5.66	0.11	49.92	0.74
	**9**	**0.88**	**0.89**	**7.73**	**0.13**	**57.56**	**0.77**
	10	0.85	0.91	9.94	0.16	60.28	0.76
	11	0.79	0.93	11.79	0.23	51.33	0.72
	12	0.70	0.95	14.20	0.32	44.76	0.65
	13	0.65	0.97	18.97	0.36	52.46	0.62
**Girls**	AUC = 0.97 (95% CI = 0.95–0.99)						
**N = 1543**	4	1.00	0.57	2.33	0.00	x	0.57
	5	1.00	0.67	3.07	0.00	x	0.67
	6	0.97	0.76	4.00	0.04	93.93	0.73
	7	0.97	0.84	6.23	0.04	163.02	0.81
	**8**	**0.97**	**0.89**	**8.76**	**0.04**	**241.62**	**0.86**
	9	0.87	0.92	10.79	0.14	76.91	0.79
	10	0.87	0.95	16.46	0.14	120.83	0.82
	11	0.81	0.96	18.48	0.20	91.29	0.76
	12	0.81	0.97	32.09	0.20	161.62	0.78

Note: AUC = area under the curve; 95%CI = 95% confidence interval; sens = sensitivity; spec = specificity;

LHR^+^ = likelihood ratio positive test; LHR*^−^* = likelihood ratio negative test; OR = diagnostic odds ratio; J = Youden's index.

All AUC's were significant (p<0.001). Scores with the highest unrounded Youden's index are indicated in bold.

The scores in the general population with the highest Youden index as cutpoints for the Problem and Competence scale and Autism score yielded concern level of ASD of respectively 16.1%, 10.1% and 6.9% children.

## Discussion

The present study evaluated the screening accuracy of the Problem and Competence scales and the newly calculated Autism score for a community sample in comparison to a sample that consists of children with an ASD diagnosis. Our results indicate that the Problem and Competence scales and the Autism score have high screening accuracy to detect ASD (i.e. AUC>0.90).

In our study we present the sensitivity and specificity for a range of BITSEA scores, because different cutpoints might be chosen in different settings (e.g. clinical application versus epidemiological research). For the comparison of the sensitivity and specificity with results of other studies we chose to discuss the sensitivity and specificity for the optimal cutpoint as indicated by the Youden index. In comparison with the prior Dutch study [27] on the screening accuracy of the BITSEA Problem scale with regard a CBCL Total Problem score in the clinical range, we found similar results; also a AUC>0.90 and no differences between subgroups. Multiple values for sensitivity and specificity of the BITSEA are reported in the study conducted in the US, because different indicators were used to classify a ‘clinical group’, and also in the Turkish study, because in their study a range of BITSEA cutpoints was applied. The US-study [19] found comparable mean sensitivity and specificity for the Problem scale as in our study. However, for the Competence scale in the US-study, a lower sensitivity and slightly higher specificity were found, compared to our study. The Turkish study [26] found slightly higher mean sensitivity and lower mean specificity for the Competence scale, compared to our study. However, the different methods to determine sensitivity and specificity (i.e. different indicators of a ‘clinical group’ and different methods to determine cutpoints), make it difficult to compare results across these studies.

The Youden index yielded the same cutpoints for boys and girls on the Problem and Competence scales. These results differ from what was found in the US-study [19], where the cutpoints on the Problem scale in children aged 24–29 months differed between boys (score 14) and girls (score 13) and also differed on the Competence scale (girls, score 15; boys, score 14). The Turkish study [26] found the same cutpoint (score 12) on the Competence scale in children aged 24–35 months, for both boys and girls. These differences between studies might be attributed to different characteristics of the study populations. Also, in the Turkish study, the ASD sample size (n = 35) was much smaller compared to our ASD sample size (n = 159).

The screening accuracy of the newly calculated Autism score was equal for boys and girls, however, the scores with the highest Youden's index differed between boys (score 9) and girls (score 8). Even though the Autism score consists of less items (17 items), its screening accuracy for ASD was better for the total population than the Problem scale (31 items), but not better than the Competence scale (11 items). The Autism score is formally not a separate BITSEA scale and the findings of the present study imply that calculation of the Autism score is unnecessary when the Competence score is known. It was to be expected that the screening accuracy of the Autism score would be at least equally well as the screening accuracy of the Competence scale, since the Autism score consists of 8 of the 11 Competence items. However, the addition of the items from the Problem scale does not further improve the screening accuracy of the Autism score.

### Limitations and strengths

Our study has some limitations. First, the BITSEA scores for the ASD-sample are based on BITSEA items that were extracted from the larger pool of ITSEA items, since parents of children in the ASD-sample completed the ITSEA.

Second, as it is expected that children with *typical* development acquire more competencies with age, previous studies have found higher Competence scores in older children, compared to younger children.[19], [22]. Our community sample consisted of a homogeneous sample with regard to age (*M* = 23.7, *SD* = 0.7). Therefore, it may not be appropriate to generalise our findings on screening accuracy of the Competence scale to children of other ages.

Third, the ASD-sample differed significantly from the community sample with regard to child's gender (more boys), and age (older children). It is likely that these characteristics might have influenced mean BITSEA scale scores; previous studies have found that mean BITSEA scores for boys are less favourable [19], [22], [23] and that mean Competence scores increase with age [19], [22]. Therefore, differences in mean BITSEA scores between the community and ASD-sample might not solely be attributed to the ASD, but also to the demographic characteristics of the samples. To compensate for these differences between conditions, we applied propensity score matching post-hoc. This yielded a sample of 900 matched cases: 750 children in the community sample en 150 in the ASD-sample, with a statistically equal boy/girl ratio (community sample: 74.5% boys, ASD-sample: 80,0% boys). There was still a significant (p<0.001) difference between matched cases regarding age (community sample: M = 28.9; SD = 7.5, ASD-sample: M = 31.8; SD = 6.4), however the effect size, Cohen's d, was small; 0.38 [39]. We calculated the AUC for the ROC-curves again for the matched sample, and no significant differences (i.e. no overlapping confidence intervals) were found compared to our prior results (data not shown).

Fourth, we do not have follow-up data on the community sample with regard to an ASD diagnosis. However, since the estimated prevalence of ASD is 1% [3], [4], we may assume that 31 children out of 3127 children will receive a diagnosis of ASD. It is difficult to estimate exactly what the effect is on our results. However, if the effect would be significant (i.e. a community sample with definitely no children with ASD would lead to other results), the mean BITSEA scores of that community sample would be more favourable than in the present study. This would mean an even larger difference in BITSEA scores, compared to the ASD sample, possibly leading to larger AUC and better sensitivity and specificity than we have found in the present study. So, due to this limitation we rather underestimate than overestimate the ‘true’ results.

A strength of our study is that the analyses were performed on a large community sample and ASD-sample which adds to the power of the study. Moreover, children in the ASD-sample were diagnosed by experienced clinicians and diagnoses were based on extensive multidisciplinary diagnostic procedures.

Additionally, another strength of our study is that parents completed the questionnaire prior to receiving a diagnostic evaluation. So parents were not biased by knowledge of an ASD diagnosis when answering the questions.

### Future research

This study evaluated the screening accuracy of the BITSEA for ASD specifically. We recommend future studies to evaluate the screening accuracy of the BITSEA for a broader range of psychosocial problems.

## Conclusions

Both the Problem and Competence scales and the Autism score have a good screening accuracy with regard to ASD for the total population and for boys and girls separately. The Autism score does not have added value to the already existing Competence score; for the screening of ASD, the Competence score is just as effective as the Autism score. Furthermore, the BITSEA is a short questionnaire and has in earlier research shown to have good reliability and validity. As mentioned before, in the introduction, early detection instruments that are used in preventive health care should cover a broad range of psychosocial problems. The BITSEA might therefore precede more extensive evaluations on ASD with other instruments, (e.g. the M-CHAT), by more specialized mental health care providers, when scores on the BITSEA indicate concern for ASD. The results of this study indicate that the BITSEA is suitable for use in the setting of (preventive) child health care for the early identification of ASD.
